# Association between herpes zoster and Parkinson’s disease and dementia: a systematic review and meta-analysis

**DOI:** 10.3389/fneur.2024.1471736

**Published:** 2024-12-05

**Authors:** Yanfeng Zhang, Weiping Liu, Yang Xu

**Affiliations:** ^1^Department of Dermatology, Tangshan Fengnan Hospital of Traditional Chinese Medicine, Tangshan, China; ^2^Department of Dermatology, First Teaching Hospital of Tianjin University of Traditional Chinese Medicine, Tianjin, China; ^3^National Clinical Research Center for Chinese Medicine Acupuncture and Moxibustion, Tianjin, China

**Keywords:** Parkinson’s disease, dementia, meta-analysis, herpes zoster, systematic review, vascular dementia

## Abstract

**Objectives:**

This meta-analysis investigated the relationship between herpes zoster and the risk of dementia or Parkinson’s disease by analyzing published clinical studies.

**Methods:**

We systematically searched PubMed, Cochrane, Embase, and Web of Science Core Collection databases on April 25, 2024. Hazard ratios (HR) were used for statistical analyses. Random-effects models were applied, and heterogeneity was assessed using the I^2^ statistic.

**Results:**

Herpes zoster was associated with a non-significant trend toward increased dementia risk (HR = 1.11, 95% CI 0.99–1.24, *p* = 0.07) but significantly increased Parkinson’s disease risk (HR = 1.15, 95% CI 1.03–1.30, *p* = 0.02). Subgroup analyses revealed that herpes zoster significantly elevated the risk of the prospective study subgroup (HR = 1.08, 95% CI 1.02–1.13, *p* = 0.004) and vascular dementia subgroup (HR = 1.17, 95% CI 1.00–1.37, *p* = 0.05). Significant heterogeneity was observed for both outcomes (dementia: *I*^2^ = 98%, *p* < 0.00001; Parkinson’s disease: *I*^2^ = 94%, *p* < 0.00001).

**Conclusion:**

Herpes zoster raises the risk of Parkinson’s disease and vascular dementia, with a potential causal link to dementia. Early vaccination against herpes zoster is recommended over post-infection antiviral treatment to mitigate risks.

**Systematic review registration:**

https://www.crd.york.ac.uk/PROSPERO/ and our registration number is CRD42024555620.

## Introduction

With the global population increasing and aging, neurodegenerative diseases, particularly Parkinson’s disease (PD) and dementia, have become significant causes of disability worldwide, posing a substantial public health burden. PD is a neurodegenerative movement disorder strongly associated with aging, with a lifetime prevalence of 1–5%, and its risk increases significantly with age. In addition to motor symptoms such as bradykinesia and resting tremor, Parkinson’s also leads to non-motor symptoms like depression, sleep disturbances, and cognitive deficits, all of which severely impact patients’ quality of life. Similarly, dementia, including Alzheimer’s disease, vascular dementia, and dementia with Lewy bodies, is an irreversible, progressive brain disorder primarily characterized by persistent cognitive impairment, significantly disrupting daily life. According to the 2015 World Alzheimer’s Disease Report, the global population of people living with dementia reached 46.8 million and is projected to double every 20 years (1–5).

Aging, genetic predisposition, educational level, and socioeconomic status are widely recognized as potential risk factors for dementia and PD (6). Emerging evidence suggests a significant correlation between neuroviral infections, accelerated brain aging, and heightened susceptibility to neurodegenerative diseases (7, 8). A variety of viruses can trigger neuroinfections, including Herpesviridae family members [e.g., Epstein–Barr virus (EBV), herpes simplex virus-1 (HSV-1), varicella zoster virus (VZV)], hepatitis C virus (HCV), human immunodeficiency virus (HIV), respiratory syncytial virus (RSV), and human endogenous retroviruses (HERVs) (9–14). Among these, more than 95% of individuals over the age of 50 globally have been exposed to VZV and the risk for this group is significant (15), necessitating further investigation into its association with dementia and PD.

A number of prospective or retrospective clinical studies have examined the relationship between herpes zoster and dementia or PD, yet the findings have been inconsistent. For instance, Cheng et al. (16) and Lai et al. (17) found an increased risk of PD in patients with herpes zoster, while Tunnicliffe et al. (18) reached the opposite conclusion. Similarly, studies investigating the relationship between herpes patients and dementia (19–21) have also yielded contradictory results. To better understand the relationship between herpes zoster and dementia or PD, we collected, analyzed, and summarized data from a wide range of studies across four commonly used databases.

## Materials and methods

### Literature search

This study followed the PRISMA (Preferred Reporting Items for Systematic Reviews and Meta-Analyses) (Supplementary Table S1) checklist published in 2020 (22) and was registered in the PROSPERO (CRD42024555620) system. We conducted a systematic literature search in four databases: PubMed, Cochrane, Embase, and Web of Science Core Collection, with a cut-off date of April 25, 2024. The search was performed in English using the keywords “herpes zoster,” “Parkinson’s disease,” and “dementia” (Supplementary Table S2). At least two clinicians manually reviewed all relevant literature multiple times to ensure relevance, eliminate disagreements, and meet the requirements.

### Inclusion and exclusion criteria

The following PICOS principles guided the inclusion criteria:

Participants: Patients with herpes zoster and a control population without herpes zoster;

Intervention: Herpes zoster;

Comparison: Control population without herpes zoster;

Outcome: Prevalence of dementia and PD (calculated as hazard ratio);

Study design: Cohort or case–control studies.

Studies were excluded if they (1) investigated infectious diseases caused by herpes viruses other than herpes zoster, (2) did not provide accessible data on the prevalence of dementia or PD, (3) were non-original papers (e.g., conference abstracts, letters, editorials, or replies), (4) were reviews, case reports, or similar, or (5) were not published in English.

### Data extraction

Yanfeng Zhang and Weiping Liu independently conducted the literature search, content screening, data extraction, and risk of bias assessment. Disagreements were resolved through consultation with a more experienced third author, Yang Xu, and consensus decision-making. Studies meeting the inclusion criteria and reporting data were included in the analyses, and outcome data related to study characteristics were extracted. Notably, despite being a conference abstract, Chen et al. (23) provided the required data for analysis and was included to collect a larger number of studies.

### Quality assessment

The Newcastle-Ottawa Scale (NOS) was used to independently assess the included case–control and cohort studies. Studies scoring 7–9 were considered high quality (24). As with data extraction, disagreements were resolved through discussion.

### Statistical analysis

Meta-analyses were performed using Review Manager version 5.4.1 (Cochrane Collaboration, Oxford, UK), with HR as the uniform assessment measure. We calculated 95% confidence intervals (95% CI) for all outcome metrics and estimated heterogeneity between studies using the inconsistency index (I^2^) (25). Significant heterogeneity was defined as *p* < 0.05 or *I*^2^ > 50%. All data were analyzed using a random effects model. For analyses with ≥10 studies, funnel plots were created using Review Manager 5.4.1, and potential publication bias was evaluated using Egger’s regression tests with Stata version 15.0 (Stata Corp, College Station, Texas, United States). *p*-values < 0.05 were considered statistically significant.

## Results

### Study characteristics and results of the screening process

**Figure 1:**
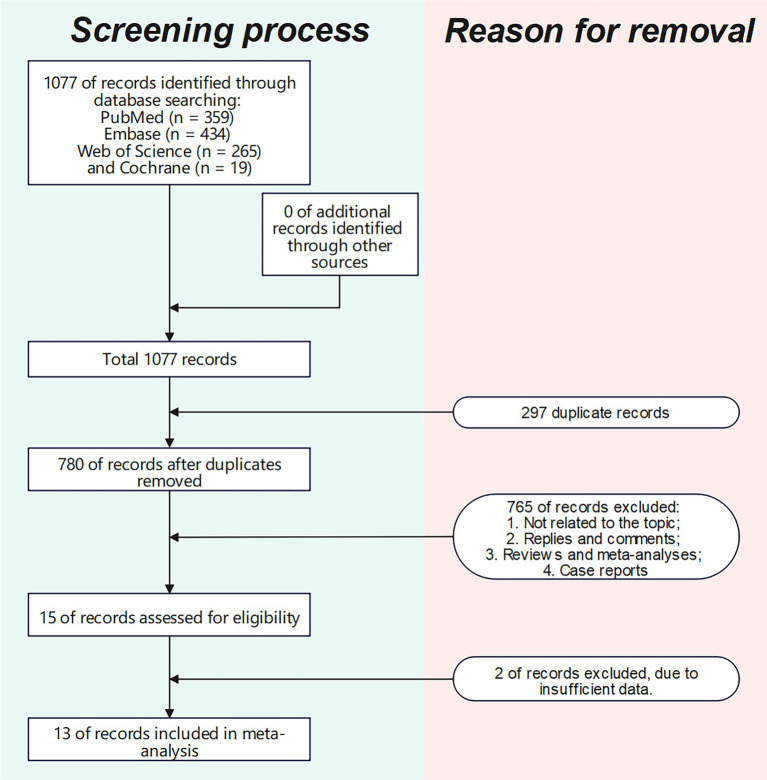
Flowchart of literature screening process.

The literature screening methodology and process are displayed in Figure 1. We searched 1,077 publications: 359 from PubMed, 434 from Embase, 19 from Cochrane, and 265 from Web of Science Core Collection. After excluding publications not meeting the inclusion and exclusion criteria, 13 studies (16–21, 23, 26–31) were included for analysis. The basic characteristics of each study are presented in Table 1.

**Table 1:** Baseline characteristics of include studies and methodological assessment.

Authors	Study period	Country	Study design	Patients (*n*)	Follow-up	Mean age (year)	Male	NOS score
				HZ/Non-HZ		HZ/Non-HZ	HZ/Non-HZ	
Bae 2021	2002–2013	Korea	Prospective cohort	34,505/195,089	11 years	60.4	13,526	7
Camacho-Soto 2020	2004–2009	USA	Case–control	14,508/193,377	NA	77.35	95,495	6
Chen 2018	1997–2013	China	Prospective cohort	39,205/39,205	NA	NA	NA	7
Cheng 2020	1998–2011	China	Prospective cohort	13,083/52,332	12.5 years	60.33	5,834/23,336	7
Choi 2021	1989–2002	Korea	Case–control	4,857/52,368	NA	NA	18,330	8
Lai 2017	1998–2010	China	Retrospective cohort	10,296/39,405	NA	74.4/73.7	5,140/19,666	6
Schmidt 2022	1997–2017	Denmark	Prospective cohort	247,305/1,235,890	21 years	64	97,509/487,464	7
Shim 2022	2010–2018	Korea	Prospective cohort	97,323/183,779	5.15 years	63.48/61.95	38,193/89,900	7
Shin 2024	2006–2017	Korea	Retrospective cohort	184,331/567,874	10.85 years	58.8	348,125	7
Tsai 2017	2001–2008	China	Retrospective cohort	846/2,538	5 years	62.2	420/1,318	9
Tunnicliffe 2024	2008–2018	USA	Prospective cohort	198,099/976,660	4.2 years	68.17/68.14	185,902/918,407	7
Warren-Gash 2022	2000–2017	UK	Retrospective cohort	177,144/706,901	5.5 years	65.1	70,690/282,061	7
Weinmann 2024	2000–2019	USA	Retrospective cohort	25,332/75,996	6.2 years	64.0	9,776/29,328	7

### Assessment of study quality

The quality scores of the included studies are shown in Supplementary Table S3. One study (30) scored 9, one (20) scored 8, nine (16, 18, 19, 21, 23, 27–29, 31) scored 7, and two (17, 26) scored 6.

### Outcomes of meta-analysis

#### Results of overall analysis

**Figure 2:**
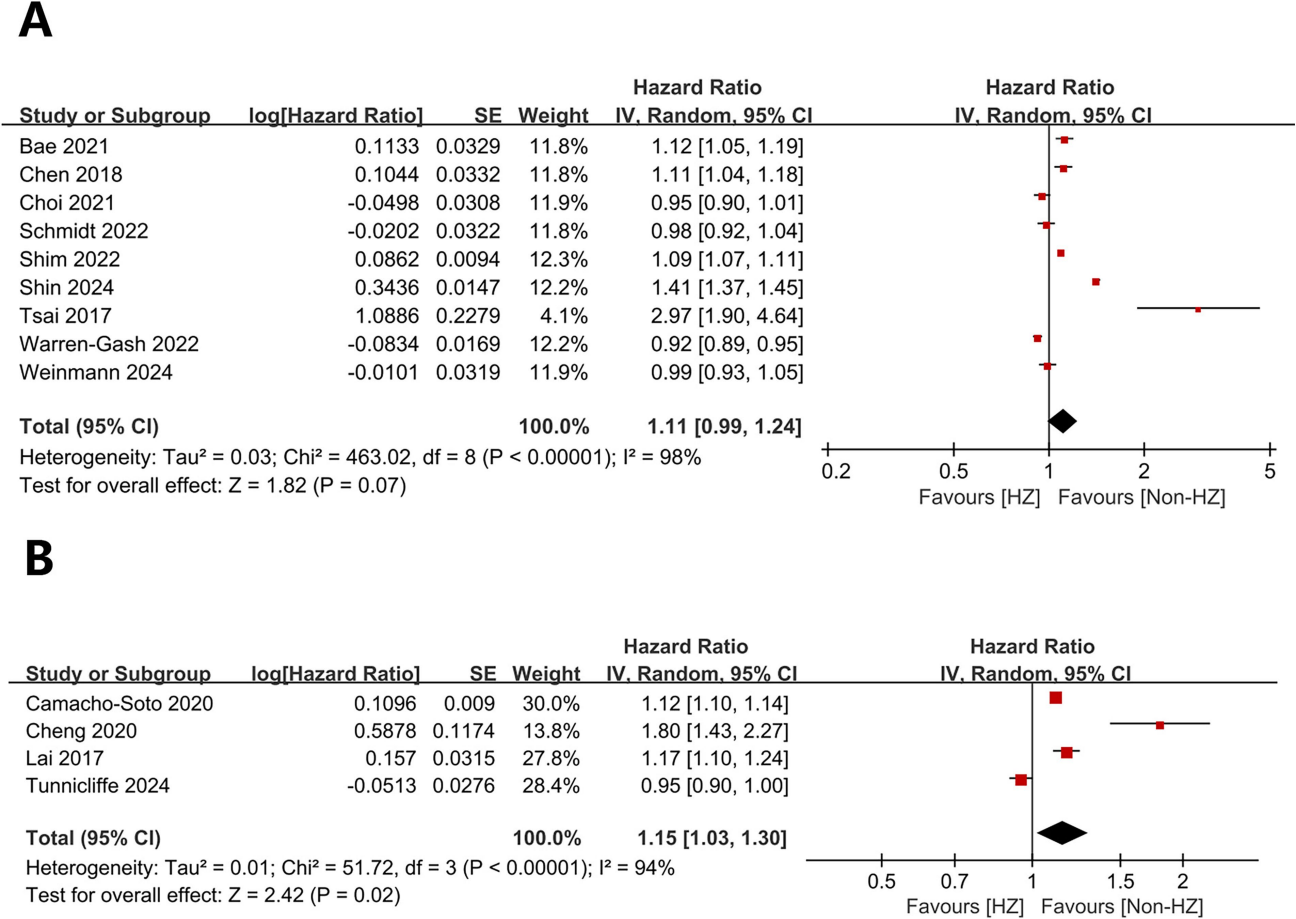
Forest plot of overall analysis results. **(A)** Forest plots for overall dementia-related analyses; **(B)** Forest plots for overall analyses related to Parkinson’s disease.

Herpes zoster did not significantly affect dementia (HR = 1.11, 95% CI 0.99–1.24, *p* = 0.07; Figure 2A) but significantly affected PD (HR = 1.15, 95% CI 1.03–1.30, *p* = 0.02; Figure 2B). Heterogeneity was present for both outcomes (dementia: *I*^2^ = 98%, *p* < 0.00001; PD: *I*^2^ = 94%, *p* < 0.00001).

#### Results of subgroup analyses

Subgroup analyses were conducted for the risk of herpes zoster and dementia but not for herpes zoster and PD due to limited data. Subgroup analyses demonstrated no significant relationships (*p* > 0.05) except for prospective studies (*p* = 0.004) in the study type subgroup. Notably, the *p*-value for vascular dementia in the dementia type subgroup was 0.05, suggesting a potential association between the variables. Heterogeneity was present in all subgroups (*I*^2^ > 50%) (Table 2).

**Table 2:** Subgroup analysis of HZ and the risk of dementia.

Subgroup	HZ and the risk of dementia			
	Study	HR [95%CI]	*p* value	*I*^2^
Total	9	1.11 [0.99–1.24]	0.07	98%
**Study design**				
Prospective	4	1.08 [1.02–1.13]	0.004	74%
Retrospective	5	1.20 [0.94–1.51]	0.14	99%
**Follow-up**				
>10 years	3	1.16 [0.92–1.47]	0.22	98%
<10 years	4	1.08 [0.94–1.23]	0.28	97%
**Sample size**				
>200,000	5	1.09 [0.94–1.27]	0.25	99%
<200,000	4	1.10 [0.96–1.27]	0.18	91%
**Age**				
50–59	2	0.64 [0.21–2.00]	0.44	90%
60–69	2	0.85 [0.53–1.37]	0.51	92%
>70	2	1.08 [0.96–1.23]	0.21	84%
**Types of dementia**				
Alzheimer’s disease	3	1.23 [0.99–1.53]	0.06	99%
Vascular dementia	3	1.17 [1.00–1.37]	0.05	79%

#### Sensitivity analysis

**Figure 3:**
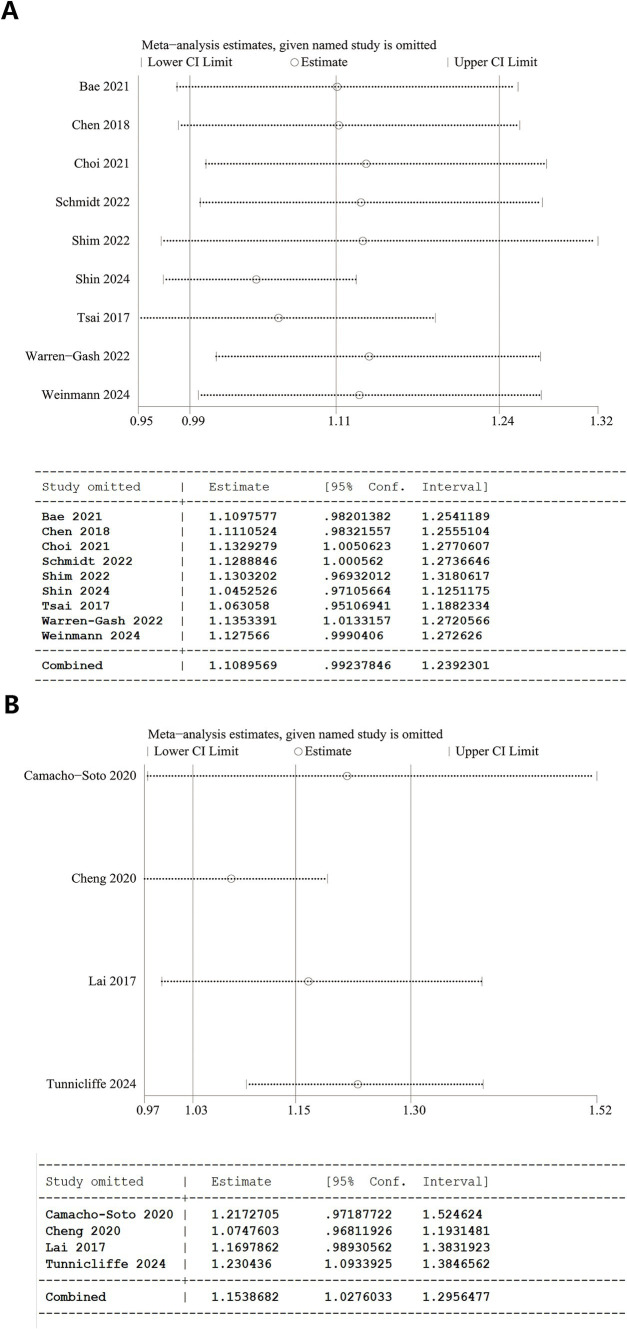
Sensitivity analysis results. **(A)** Overall analysis of herpes zoster and dementia and 95% CI calculations from Stata software. **(B)** Overall analysis of herpes zoster and Parkinson’s disease and 95% CI calculations from Stata software.

A one-way sensitivity analysis demonstrated the instability of both overall analyses. In the analyses of herpes zoster and dementia, the overall results were destabilized if studies by Choi et al. (20), Schmidt et al. (27), or Warren-Gash et al. (19) were individually excluded. Similarly, when evaluating herpes zoster and PD, the results became unstable with the removal of Camacho-Soto et al. (26), Cheng et al. (16), or Lai et al. (17) (Figure 3).

## Discussion

The potential link between nervous system infections and neurodegeneration has gained widespread attention since Bowery et al.’s pioneering study in 1992, which demonstrated tetanus toxin-induced neurodegeneration in rats (32, 33). A variety of viruses that can cause infections in the nervous system [e.g., herpesvirus family (EBV, HSV, VZV, etc.), HCV, HIV, RSV, etc.] may cause protein aggregation, abnormalities in energy balance, and inflammation and lead to neurodegenerative pathologies. In recent years, viral stimulation of microglia activation in neurodegeneration has become a hot topic again in the context of SARS-CoV-2 and COVID-19. Given that over 95% of individuals aged 50 and older globally have been exposed to varicella-zoster virus, a large portion of the population is at risk for herpes zoster (15). Its potential association with dementia and PD is therefore a relevant topic. Due to the many unresolved physiological mechanisms and the ongoing controversy in current clinical study conclusions, we collected and reviewed as much relevant literature as possible to better understand the association between herpes zoster and these two neurodegenerative diseases.

Regarding dementia, our comprehensive analysis results showed that although a positive trend was observed between herpes zoster and dementia, this association lacked statistical significance. Dementia encompasses various types, such as Alzheimer’s disease, vascular dementia, dementia with Lewy bodies, frontotemporal dementia, Huntington’s disease, and Creutzfeldt-Jakob disease, each with distinct pathogenic mechanisms and marker factors. Herpes zoster may influence only certain types of dementia. To further investigate the potential link, we thoroughly reviewed the data from the included studies and performed subgroup analyses based on five factors: study type, follow-up duration, sample size, subject age, and dementia classification.

The subgroup analysis revealed that factors such as follow-up duration, sample size, and participant age did not significantly affect the results. However, prospective studies demonstrated a clear positive association between herpes zoster and the risk of dementia, while retrospective studies showed no such significance. Notably, in the sensitivity analysis, excluding a larger number of retrospective studies led to a trend indicating an increased risk of dementia linked to herpes zoster.

This suggests a potential causal relationship between the two. More rigorous methods are needed to determine causality, considering confounding factors. Recent advances in evidence-based medicine have enabled Mendelian randomization to offer a more robust approach to causal inference. A recent Mendelian randomization study on the causal relationship between herpes zoster and dementia supports this conclusion, consistent with findings from a subgroup of prospective studies (34).

In a subgroup analysis of dementia types, herpes zoster was found to significantly increase the risk of vascular dementia, while the effect on Alzheimer’s disease, although showing an upward trend, did not reach statistical significance. This observation may warrant a mechanistic explanation. Although the mechanisms by which viruses cause neurodegenerative diseases remain poorly understood, based on available studies, the significant increase in the risk of vascular dementia may result from primary varicella-zoster virus infection, which initially causes chickenpox and subsequently remains latent. Under conditions such as aging or immunosuppression, the virus may reactivate and trigger herpes zoster. During this process, VZV may invade the central nervous system, causing vasculopathy and impairing cerebral blood flow, which can result in cerebral infarction and vascular dementia (35–40). In Alzheimer’s disease, neuroinflammation caused by the abnormal accumulation of amyloid β (Aβ) peptide and tau protein is the main pathogenesis. Systemic inflammation, triggered by viral infections and microglial activation, exacerbates Aβ and tau protein accumulation, thereby promoting Alzheimer’s disease progression (41, 42) Moreover, Aβ is not only a key pathological protein in Alzheimer’s disease but also a potential cellular receptor for VZV (43). The presence of Aβ may interfere with VZV replication, partially protecting the host against viral infection. This mechanism could lead to a gradual accumulation of Aβ during Alzheimer’s disease progression and a reduction in VZV’s effects (44–47) potentially explaining the nonsignificant association with herpes zoster despite a trend of increased Alzheimer’s disease risk.

Our analysis revealed a significant increase in the risk of developing PD following herpes zoster infection. However, due to the limited data in existing studies, a more detailed subgroup analysis could not be performed. PD is a complex neurodegenerative disorder presenting both motor and non-motor symptoms. The hallmark pathological features include the loss of dopaminergic neurons (48) and abnormal α-synuclein aggregation (49). The reduction of dopaminergic neurons primarily contributes to motor symptoms such as resting tremor, muscle rigidity, and bradykinesia, whereas non-motor symptoms, including cognitive impairment, autonomic dysfunction, and neurobehavioral abnormalities, are linked to α-synuclein aggregation (50). There is currently insufficient research to directly clarify the relationship between VZV and the dopamine system. Consequently, we concentrate on the non-motor symptoms of PD, particularly Parkinson’s dementia. While no definitive study has yet identified the exact mechanism through which VZV contributes to Parkinson’s dementia, we hypothesize that herpes zoster might increase the risk through the following mechanisms: abnormal aggregation of α-synuclein is a key pathological process in PD (51, 52). VZV may influence α-synuclein expression, a protein crucial to PD pathogenesis, by inducing vasculopathy, which impairs α-synuclein clearance and results in its abnormal accumulation in the brain, thereby promoting PD development (53, 54). Cross-reactivity between α-synuclein and herpesvirus peptides has been observed in PD patients (55). Abnormal α-synuclein aggregation is not limited to PD but is also linked to other α-synucleinopathies, including dementia with Lewy bodies, multiple system atrophy, the Lewy body variant of Alzheimer’s disease, and pure autonomic failure. Cognitive impairment in these conditions often accompanies cerebrovascular-like diseases (56), and VZV infection can induce vasculopathy. Thus, a theoretical association between VZV infection and other α-synucleinopathies may exist, though this hypothesis remains infrequently explored and requires further investigation.

Two key aspects of VZV infection and neurodegenerative diseases warrant attention. First, HERV-DNA transposable elements that constitute about 8% of the human genome—play a crucial role (14). Studies suggest a synergistic effect of HERVs and VZV in the pathogenesis of multiple sclerosis, potentially accelerating disease progression (57). The role of HERVs in conjunction with VZV in major neurodegenerative diseases, such as Alzheimer’s and PD, remains inadequately explored, with numerous aspects still uncharted. The mitochondrial dysfunction hypothesis provides new insights into neurodegenerative disease mechanisms. Recent theories suggest that viruses might expedite microglial aging and facilitate neurodegenerative disease progression by activating microglia and inducing mitochondrial dysfunction. Moreover, VZV infection has been shown to alter mitochondrial morphology, causing fragmentation and swelling. These mitochondrial abnormalities may result in cellular damage or death during infection, thereby contributing to the pathogenesis of neurodegenerative diseases (58).

Given the risks associated with herpes zoster, neurodegenerative diseases, and the significant impact of postherpetic neuralgia (PHN) on patients’ quality of life, appropriate coping strategies are crucial. Antiviral drugs are commonly used to treat herpes zoster. Among these, acyclovir (including its derivatives such as valaciclovir and famciclovir) is often the preferred choice. Evidence from clinical practice suggests that early antiviral therapy may lead to better outcomes. This is supported by some studies (59). It is important to note that the use of antiviral drugs is limited by various factors, including financial capacity, medical conditions, and individual health status, leading to differences in treatment outcomes. Among the 13 papers analyzed, four focused on antiviral therapy. Two studies did not find significant antiviral treatment effects after accounting for factors such as BMI, comorbidities, and unhealthy habits, while the other two suggested a potential protective effect. These findings highlight the ongoing controversy surrounding the role of antiviral therapy in reducing the risk of herpes zoster in patients with two neurodegenerative diseases.

We consider timely vaccination, especially in the absence of disease, to be the most optimal strategy at present. Various herpes zoster vaccines, including Mosquirix, Shingrix, and Nuvaxovid, are available on the market. Notably, Shingrix, the most effective vaccine, shows efficacy ranging from 96.6 to 97.9% across all age groups, with an overall efficacy of 97.2% (60). Furthermore, recently developed vaccines based on multi-nanoparticle (NP) platforms have achieved superior protective efficacy (61).

In reviewing our study, we must face up to its limitations, which are critical to fully understand and accurately assess the potential impact of the relationship between herpes zoster and dementia and PD. Firstly, despite our best efforts to include a large number of studies and to strictly control for inter-data variability factors, the high heterogeneity of the data remains a problem that cannot be ignored. This heterogeneity stems mainly from sample size limitations, which prevented certain in-depth subgroup analyses from being conducted or, if they were conducted, made it difficult to produce results with low heterogeneity. Therefore, we are cautious about the findings obtained and recognize that they may need to be further validated in larger, more refined studies in the future. Secondly, as our study primarily focused on older individuals, the applicability to younger age groups should be interpreted with caution. Further, some known risk factors for dementia or Parkinson’s, such as genetic factors, alcohol abuse, smoking, exposure to pesticides, or use of well water, were not comprehensively documented in the data from all studies in our study. This lack of information may have led to some bias in our findings, which do not fully and accurately reflect the impact of these potential factors on disease risk. Finally, our study did not break down the location of the appearance of herpes zoster. It has been shown that the association between VZV infection in the oral cavity and eyes and the risk of dementia is much stronger (29). Also, the different locations of herpes zoster may affect the diagnostic accuracy of physicians and the motivation of patients, which in turn may have an impact on the prevention and control of the disease. This factor was not fully considered in our study and may also be a reason for the biased results.

## Conclusion

In conclusion, based on a comprehensive review of 13 relevant studies, we investigated the association between herpes zoster and dementia or PD. The findings indicate that herpes zoster significantly raises the risk of PD and vascular dementia. Additionally, a causal relationship exists between herpes zoster infection and dementia. Early vaccination against herpes zoster is recommended to mitigate risks, rather than antiviral treatment post-infection.

## Data Availability

The original contributions presented in the study are included in the article/Supplementary material, further inquiries can be directed to the corresponding author.
