# Supplementary material for: Association between herpes zoster and Parkinson’s disease and dementia: a systematic review and meta-analysis
Source: Front Neurol. 2024 Dec 5;15:1471736. doi: 10.3389/fneur.2024.1471736 (PMC11655326; doi:10.3389/fneur.2024.1471736)
Supplement: Supplementary file 2 [file Table_2.docx]

Supplementary Table S2. Search strategy

| Pubmed 359 articles：  (("Herpes Zoster"[Mesh]) OR (((Zona[Title/Abstract]) OR (Zoster[Title/Abstract])) OR (Shingles[Title/Abstract]))) AND ((("Parkinson Disease"[Mesh]) OR (((((((Idiopathic Parkinson's Disease[Title/Abstract]) OR (Lewy Body Parkinson's Disease[Title/Abstract])) OR (Parkinson's Disease[Title/Abstract])) OR (Idiopathic Parkinson Disease[Title/Abstract])) OR (Lewy Body Parkinson Disease[Title/Abstract])) OR (Primary Parkinsonism[Title/Abstract])) OR (Paralysis Agitans[Title/Abstract]))) OR (("Dementia"[Mesh]) OR (((Dementias[Title/Abstract]) OR (Amentia[Title/Abstract])) OR (Amentias[Title/Abstract])))) |
| --- |
| Embase 434 articles：  ((Herpes Zoster or (Zona or Zoster or Shingles)) and (Parkinson Disease or (Idiopathic Parkinson's Disease or Lewy Body Parkinson's Disease or Parkinson's Disease or Idiopathic Parkinson Disease or Lewy Body Parkinson Disease or Primary Parkinsonism or Paralysis Agitans) or (Dementia or (Dementias or Amentia or Amentias)))).ab. |
| Cochrane 19 articles：  ((Herpes Zoster or (Zona or Zoster or Shingles)) and (Parkinson Disease or (Idiopathic Parkinson's Disease or Lewy Body Parkinson's Disease or Parkinson's Disease or Idiopathic Parkinson Disease or Lewy Body Parkinson Disease or Primary Parkinsonism or Paralysis Agitans) or (Dementia or (Dementias or Amentia or Amentias)))).ab. |
| Web of science collection 265 articles：  ((Herpes Zoster) OR (((Zona) OR (Zoster)) OR (Shingles))) AND (((Parkinson Disease) OR (((((((Idiopathic Parkinson's Disease) OR (Lewy Body Parkinson's Disease)) OR (Parkinson's Disease)) OR (Idiopathic Parkinson Disease)) OR (Lewy Body Parkinson Disease)) OR (Primary Parkinsonism)) OR (Paralysis Agitans))) OR ((Dementia) OR (((Dementias) OR (Amentia)) OR (Amentias)))) (Abstract) |
