# Supplementary material for: Association between herpes zoster and Parkinson’s disease and dementia: a systematic review and meta-analysis
Source: Front Neurol. 2024 Dec 5;15:1471736. doi: 10.3389/fneur.2024.1471736 (PMC11655326; doi:10.3389/fneur.2024.1471736)
Supplement: Supplementary file 3 [file Table_3.docx]

| Supplementary Table S3. Quality evaluation of the eligible studies with Newcastle–Ottawa scale. | | | | | | | | | |
| --- | --- | --- | --- | --- | --- | --- | --- | --- | --- |
| Study | Selection | | | | Comparability | | Outcome | | |
|  | Representative-ness | Selection of  non-exposed | Ascertainment  of exposure | Outcome not present at start | Comparability on most important factors | Comparability on other risk factors | Assessment of outcome | Long enough follow-up (median≥1 year) | Adequacy  (completeness) of follow-up |
| Bae 2021 | * | * | * | * | - | - | * | * | * |
| Camacho-Soto 2020 | - | * | * | * | - | - | * | * | * |
| Chen 2018 | * | * | * | * | - | - | * | * | * |
| Cheng 2020 | * | * | * | * | - | - | * | * | * |
| Choi 2021 | * | * | * | * | * | - | * | * | * |
| Lai 2017 | - | * | * | * | - | - | * | * | * |
| Schmidt 2022 | * | * | * | * | - | - | * | * | * |
| Shim 2022 | * | * | * | * | - | - | * | * | * |
| Shin 2024 | * | * | * | * | - | - | * | * | * |
| Tsai 2017 | * | * | * | * | * | * | * | * | * |
| Tunnicliffe 2024 | * | * | * | * | - | - | * | * | * |
| Warren-Gash 2022 | * | * | * | * | - | - | * | * | * |
| Weinmann 2024 | * | * | * | * | - | - | * | * | * |
| *indicates criterion met; - indicates significant of criterion not met. | | | | | | | | | |
